# Anticoagulation, recurrent thrombosis, and major bleeding in antiphospholipid syndrome: UK multicenter observational study

**DOI:** 10.1182/bloodadvances.2025019447

**Published:** 2026-02-24

**Authors:** Christina Crossette-Thambiah, Indika Rajakaruna, Zain Odho, Andrew J. Doyle, Karen A. Breen, Michael Laffan, Deepa J. Arachchillage, HaemSTAR Study Collaborators, Giulia Simini, Lara Roberts, Amna Gameil, Gillian Lamb, Kay Simpson, Nini Aung, Li Yuan Chan, Emily Booth, Ann Benton, James Duffell, Kieron Power, Saniya Dhawan, Izabela James, Sarah Lewis, Jessica Anderson, Sahil Bhagat, Mohammed Altohami, Amjad Hmaid, Dina Abuqamar, Pedro Goncalves, Zunaid Chunara, Styliani Salta, Amy Webster, Sarah Challenor, Pip Nicholson, David Sutton, Richard Buka, Angel Joseph, Victor Ling, Jacob Nkem, Kat Moss, Andrew Ross, Emily Millen, Alexander Bashford, Prajakta Pardeshi, Philip Mounter, Juan Tan, Cecilia Gyansah, Tina Dutt, Samuel Badu, Francesca Murphy, Alexander Langridge, Mohammad Osama, Paula Glancy, Shikha Chattree, Alexander Langridge, Kathryn Musgrave, Beatrice Likupe, Alison Delaney

**Affiliations:** 1 Centre for Haematology, Department of Immunology and Inflammation, Imperial College London, London, United Kingdom; 2 Department of Haematology, Imperial College Healthcare NHS Trust, London, United Kingdom; 3 Department of Computer Science and Digital Technologies, University of East London, London, United Kingdom; 4 Haemostasis and Thrombosis Centre, Guy’s and St Thomas’ NHS Foundation Trust, London, United Kingdom

## Abstract

Antiphospholipid syndrome (APS) is an acquired autoimmune disease characterized by the presence of thrombosis and/or pregnancy morbidity with persistently positive antiphospholipid antibodies (aPL). Due to the heterogeneity and relative rarity of this disorder, clinical practice in the management of APS remains varied. In this retrospective UK-wide multicenter study, we aimed to delineate clinical practice and outcomes in thrombotic APS to improve the areas of limited knowledge, particularly anticoagulant practice, thrombotic recurrence, and bleeding. Anticoagulation in Antiphospholipid Syndrome is the largest multicenter observational study to date, spanning 20 National Health Service trusts in the UK, and including 500 adult patients (aged ≥18 years) with thrombotic APS, on or off anticoagulation during 2012-2021. Thrombotic APS is primarily treated with vitamin K antagonists; however, the rates of recurrent thrombosis remain high. In the last decade, recurrent thrombosis occurred in 43 of 125 (34.4%), 31 of 95 (32.6%), and 37 of 93 (39.8%) patients with single-, dual-, and triple-positive aPL, respectively, with a recurrence rate of 46%. There was no difference in the probability of recurrent thrombosis based on the number of positive aPL tests (*P* = .82), especially in the first 3 years. However, the probability of recurrent thrombosis was significantly higher in patients with lupus anticoagulant (*P* < .01) compared with the presence of other antibodies. There was a higher probability of recurrence in patients with arterial than venous thrombosis (*P* = .03). Overall, the 10-year bleeding rate was 22.0%, with 6.7% of patients having major bleeding. Identifying patients with APS at higher risk of recurrent thrombosis remains a challenge, and current risk stratification is not adequate.

## Key Points

• Due to the heterogeneity and relative rarity of this disorder, clinical practice in the management of APS remains varied.
• The 10-year recurrent thrombosis rate was 46%, with a higher probability of recurrence in patients with lupus anticoagulant (*P* < .01).

## Visual Abstract

**Figure:**
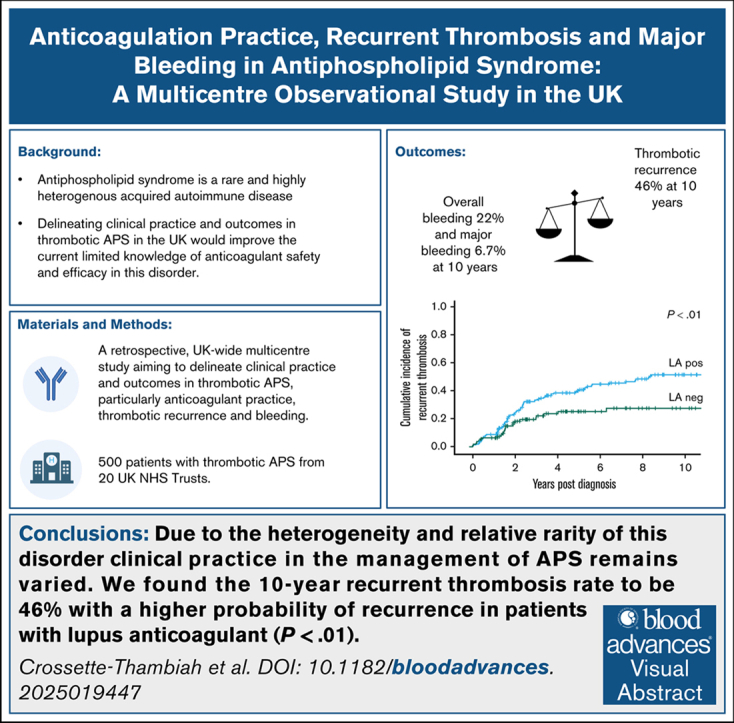

## Introduction

Antiphospholipid syndrome (APS) is an acquired autoimmune disease characterized by development of thrombosis and/or specifically defined pregnancy morbidity in association with persistently positive antiphospholipid antibodies (aPL). Various autoantibodies can be found in patients with APS, but current clinical diagnosis of APS is based on the detection of ≥1 antibodies defined as “criteria antibodies,” namely lupus anticoagulant (LA), immunoglobulin G and immunoglobulin M anti-cardiolipin antibodies (aCL), and anti–β2-glycoprotein I (anti-β2GPI) antibodies on ≥2 occasions >12 weeks apart.^1^ APS is a highly prothrombotic disease, especially in patients with triple-positive aPL (presence of all 3 criteria aPL), who are reported to be at highest risk of both a first thrombotic event and further recurrence.^2^

Despite the autoimmune nature of the disease, the current mainstay of treatment in thrombotic APS is with anticoagulation. However, there is a clear difference in the efficacy of different oral anticoagulants, notably for triple-positive aPL or patients with arterial thrombosis.^3^ Vitamin K antagonists (VKA) have been used in patients with thrombotic APS for many years, but direct-acting oral anticoagulants (DOACs) including rivaroxaban, apixaban, and edoxaban (direct Factor Xa inhibitors) and dabigatran (direct thrombin inhibitor) have been established as first-line treatment for patients with venous thrombosis (VTE) without APS, owing to their safety data, ease of administration, and lack of requirement for monitoring.^4^

Consequently, the use of DOACs in patients with APS has been investigated. The Trial of Rivaroxaban in AntiPhospholipid Syndrome, a randomized open-label multicenter noninferiority study comparing rivaroxaban with VKA in thrombotic triple-positive APS, was prematurely terminated due to excess arterial thrombotic events in patients receiving rivaroxaban (12%) compared with no recurrent thrombosis in the VKA group.^5^ Major bleeding events occurred in 7% and 3% of patients treated with rivaroxaban and VKA, respectively.^5^ This culminated in the Medicines and Healthcare Products Regulatory Agency and European Medicines Agency issuing recommendations that DOACs should not be used for treatment or secondary prevention of thrombosis in all patients with APS, especially in those with triple-positive APS.^6^^,^^7^ Subsequently, a meta-analysis of 4 open-label randomized controlled trials^3^^,^^5^^,^^8^^,^^9^ comparing outcomes in 472 patients with APS receiving DOACs or VKA showed that DOACs were associated with an increased risk of arterial thrombotic events (odds ratio [OR], 5.43 [95% confidence interval (CI), 1.87-15.75]; *P* < .001), especially stroke (OR, 10.74 [95% CI, 2.29-50.38]).^10^ Importantly, this risk persisted regardless of aPL status (ie, triple-positive or not) and regardless of index thrombotic event (VTE or arterial thrombosis). No difference in major bleeding events between 2 anticoagulant types were observed. Furthermore, there was only a trend toward a higher risk of recurrent arterial thrombosis in patients with single- or dual-positivity aPL treated with a DOAC compared with VKA (OR, 4.29 [95% CI, 0.75-24.43]).^10^ As such, the British Society for Haematology guidelines on APS in 2020, and updated in 2024, recommended against the use of DOACS, specifically following arterial thrombosis and VTE in individuals who were “triple-positive.” Furthermore, in the absence of strong evidence for superior efficacy of VKA over DOACs in these groups, these guidelines suggested, rather than recommended, the use of VKA in thrombotic APS with single- or dual-positive aPL following VTE.^6^

The prospect of further clinical trials investigating optimal therapy for patients with thrombotic APS with non–triple-positive aPL is limited due to concern over recurrent thrombosis with DOAC and also rarity of the disease. Furthermore, an important unresolved question in the management of APS is whether arterial thrombosis in patients with APS is best managed with antiplatelet agents (single or dual) alone, anticoagulant therapy alone, or by a combination of antiplatelet treatment and anticoagulant.^11^ Delineating clinical practice and outcomes in thrombotic APS in the United Kingdom would improve the current limited knowledge of anticoagulant safety and efficacy in this disorder.

In this retrospective UK-wide multicenter study, we aimed to evaluate anticoagulation practice, determine compliance with the current British Society for Haematology guidelines, and explore rates of thrombotic recurrence and bleeding in thrombotic APS.

## Materials and methods

### Study design

Anticoagulation in Antiphospholipid Syndrome is a multicenter observational study across 20 National Health Service (NHS) trusts in the United Kingdom. The study was deemed an audit and not research by the NHS Health Research Authority research decision tool. Therefore, formal ethics approval was not required. Results were compared with current British Society for Haematology recommendations retrospectively. Data collection was approved by the audit departments at each participating center before data entry (audit registration number HAE_018). Data were collected retrospectively from patient clinical records by medical professionals with no breach of privacy or anonymity and allocating a unique study number. Inclusion criteria for the study were adult patients (aged ≥18 years at the time of first thrombosis) with thrombotic APS on or off anticoagulation under clinical review between January 2012 and January 2022. Thrombotic events were objectively confirmed by radiological imaging and/or clinical review, especially transient ischemic attack. Minimal testing for APS comprised the testing for all 3 standard aPL (LA, immunoglobulin M/ immunoglobulin G aCL, and anti-β2GPI), and patients were only included in the 10-year event analysis after confirmation of aPL positivity following repeat testing at ≥12 weeks. Exclusion criteria were patients aged <18 years and patients with only obstetric APS.

### Data collection

Data collection was facilitated nationally via the Haematology Specialists in Training, Audit & Research network (HaemSTAR), a UK-wide network of clinical hematology trainees supported by the National Institute of Health Research nonmalignant clinical research network (collaborators listed in the Footnotes). This project used the secure Research Electronic Data Capture database (Research Electronic Data Capture version 10.0.10; Vanderbilt University, Nashville, TN) hosted by Imperial College London. Data were entered by clinicians responsible for patient care onto a bespoke standardized case report form. Baseline patient demographics, comorbidities, aPL status, and relevant clinical history, including thrombotic, bleeding events, and anticoagulation details, were collected.

### Outcomes

The primary outcome was to assess antithrombotic use according to type of initial thrombosis and triple vs nontriple APS type. Secondary outcomes were to assess thrombosis recurrence during follow-up according to APS testing patterns and type of antithrombotic. Major bleeding as a safety outcome was also assessed.

### Statistical analysis

Categorical variables were reported as proportion and/or percentage. Continuous variables were reported as mean (± standard deviation) or median (range) value. The Fisher exact test or χ^2^ test for categorical variables and the Student *t* test or Wilcoxon-Mann-Whitney test for continuous variables were used as appropriate. Survival analysis was performed using the Kaplan-Meier method, and the log-rank test was used to examine the differences between curves. Multivariable Cox regression models were performed to determine the ORs and 95% CIs of individual factors of interest. To identify factors associated with the probability of thrombosis-free survival, Kaplan-Meier curves were compared using the log-rank test. Factors found to be statistically significant at the *P* < .2 level were then entered into a stepwise Cox regression analysis to produce a final model that encompassed all independent prognostic factors significant at *P* < .05. Tests of significance were 2-sided, and a *P* value < .05 was considered significant. Analyses and figures were performed using R (R Foundation for Statistical Computing, Vienna, Austria, http://www.R-project.org).

Institutional Review Board has approved the study (Audit registration number HAE_018), which was conducted in accordance with the Declaration of Helsinki.

## Results

### Baseline characteristics

Five hundred patients with APS diagnosed after a first thrombosis from across 20 UK NHS Trusts were included in this study. The patients were predominantly women (58.6%), and the median age was 45.6 years (interquartile range, 18-87 years) at the time of diagnosis; 37.4% of patients had coexistent autoimmune diseases. Baseline characteristics of the group including aPL profile and drug history are summarized in Table 1.

**Table 1.** **Baseline characteristics, including demographics, aPL profile, vascular risk factors, and antithrombotic treatment of all patients, patients with venous thromboembolism and patients with arterial thrombosis**

	All thrombosis (N = 500) (%)	VTE (n = 366) (%)	Arterial thrombosis (n = 134) (%)
**Total**	500	366	134
**Sex**			
Men	207 (41.4%)	125 (34.1%)	82 (61.2%)
Women	293 (58.6%)	241 (65.8%)	52 (38.8%)
**Age (y)**			
IQR	18-88	18-76	22-88
Median	45.6	42.3	55.7
18-30	56 (11.2%)	45 (12.3%)	11 (8.2%)
31-50	160 (32.2%)	128 (35.0%)	32 (23.9%)
51-70	212 (42.4%)	164 (44.8%)	48 (35.8%)
>71	72 (14.4%)	29 (7.9%)	43 (28.4%)
**Antibody status at time of diagnosis**			
Single-positive	155 (31%)	117 (31.9%)	38 (28.4%)
Dual-positive	163 (32.6%)	114 (31.1%)	49 (36.6%)
Triple-positive	182 (36.4%)	135 (36.9%)	47 (35.1%)
**Anticoagulation type initiated**			
VKA∗	345 (69.0%)	236 (64.5%)	109 (81.3%)
DOAC	85 (17.0%)	76 (20.8%)	9 (6.7%)
Other/none	70 (14.0%)	54 (14.8%)	16 (11.9%)
**INR of those on VKA**			
2.0-3.0	278 (80.6%)	167 (85.2%)	99 (72.3%)
3.0-4.0	67 (19.4%)	29 (14.7%)	38 (27.7%)
**Coexistent autoimmune disease**			
Total	187 (37.4%)	139 (37.9%)	48 (35.8%)
SLE	72 (39.6%)	50 (35.9%)	22 (45.8%)
Rheumatoid arthritis	38 (20.3%)	18 (12.9%)	20 (41.6%)
Sjogren syndrome	15 (8.0%)	9 (6.5%)	6 (12.5%)
Hypothyroidism	10 (5.3%)	7 (5.0%)	3 (6.2%)
Other	52 (27.8%)	32 (23.0%)	20 (41.6%)
**HCQ**			
200 mg od	66 (13.2%)	37 (10.1%)	29 (21.6%)
200 mg bd	43 (8.6%)	19 (5.2%)	24 (17.9%)
**Antiplatelet therapy initiated**			
Any antiplatelet	178 (35.6%)	110 (30.1%)	68 (50.7%)
Single	147 (82.6%)	89 (80.9%)	58 (85.3%)
Dual	31 (17.4%)	21 (19.1%)	10 (14.7%)
**Arterial risk factors**			
Hypertension	212 (42.4%)	96 (27.1%)	116 (62%)
Diabetes mellitus	194 (38.8%)	99 (28%)	95 (50.8%)
IHD	50 (10%)	28 (8%)	22 (11.8%)
Hypercholesterolemia	124 (24.8%)	66 (18.6%)	58 (31%)
Smoking	109 (21.8%)	44 (12.4%)	65 (34.7%)
High BMI (>30 kg/m^2^)	64 (12.8%)	30 (8.5%)	34 (18.1%)

bd, twice a day; BMI, body mass index; IHD, ischemic heart disease; IQR, interquartile range; od, once a day; SLE, systemic lupus erythematosus; y, years.

∗ Eight of 500 (1.6%) patients were on an alternative VKA that was not warfarin owing to allergic reactions.

### Anticoagulation practice

**Figure 1.**
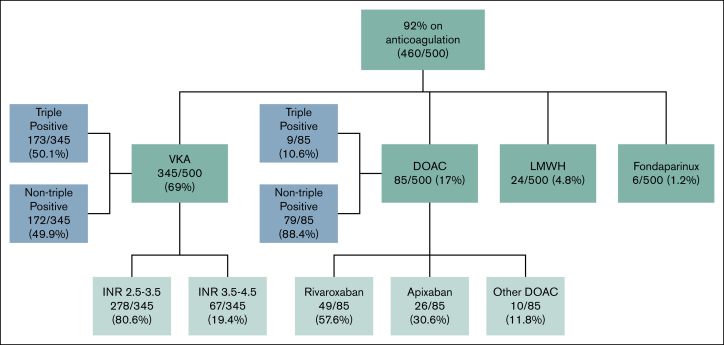
**Flowchart demonstrating anticoagulation practice in the 500 patients included in this study.** LMWH, low-molecular-weight heparin.

Overall anticoagulation practice in the study group is summarized in Figure 1.

### Thrombotic events

Overall, 366 of 500 (73.2%) patients had had VTE, and 134 of 500 (26.8%) arterial thrombosis at the time of APS diagnosis.

### Arterial thrombosis

Baseline characteristics of those with arterial thrombosis are summarized in Table 1. The sites of arterial thrombosis were stroke in 92 of 134 (68.7%), myocardial infarction in 33 of 134 (24.6%), and transient ischemic attack in 9 of 134 (6.7%) patients; 68 (50.7%) patients with arterial thrombosis were on an antiplatelet treatment in addition to anticoagulation. No patients were on antiplatelet treatment alone. On univariate analysis, increasing age at diagnosis was associated with increased risk of arterial thrombosis between ages of 40 and 60 years (OR, 4.12 [95% CI, 1.71-11.6]; *P* = .003), and increased further in those aged >60 years (OR, 7.74 [95% CI, 3.25-21.6]; *P* < .001) compared with those aged between 18 and 40 years. Additionally, men had a higher likelihood of developing arterial thrombosis (OR, 1.97 [95% CI, 1.19-3.29]; *P* = .009) on univariate analysis. Other parameters increasing the risk of arterial thrombosis on univariate analysis included smoking (OR, 3.22 [95% CI, 1.61-6.61]; *P* < .001) and hypocholesteremia (OR, 5.02 [95% CI, 29-7.09]; *P* < .001). On univariate analysis, triple-positive antibody status did not significantly affect the risk of arterial thrombosis (OR, 1.17 [95% CI, 0.69-2.0]; *P* = .6), although interestingly, double-positive aCL and anti-β2GPI was associated with increased risk of arterial thrombosis (OR, 2.04 [95% CI, 1.13-3.69]; *P* = .01). All parameters significant on univariate analysis were included in the multivariate analysis (MVA). Increased age of 40 to 60 years remained significant on MVA (OR, 4.43 [95% CI, 1.65-13.7]; *P* = .005), as did age >60 years (OR, 5.71 [95% CI, 2.09-17.8]; *P* < .001). Smoking, hypercholesterolemia, and double-positivity for aCL and anti-β2GPI also all remained positive on MVA (OR, 3.11 [95% CI, 1.36-7.36]; *P* < .008; OR, 3.62 [95% CI, 1.89-7.09]; *P* < .001; and OR, 4.01 [95% CI, 1.33-12.7]; *P* = .015, respectively). The increased risk of arterial thrombosis in men was no longer statistically significant on MVA. Taken together, those presenting with arterial thrombosis were more likely to be older men, with high cholesterol and a history of smoking, and an antibody profile of dual aCL and anti-β2GPI positivity.

### Venous thrombosis

Baseline characteristics of those with VTE are summarized in Table 1. The sites of VTE were deep vein thrombosis in 156 of 366 (42.6%) patients, cerebral venous sinus thrombosis in 100 of 366 (27.3%) patients, pulmonary embolism in 95 of 366 (25.9%) patients, and portal vein thrombosis in 15 of 366 (4.1%) patients. Unlike arterial thrombosis, age, atherosclerotic risk factors, or any particular antibody profile were not associated with VTE, suggesting they may be a more heterogeneous group or more dependent on circumstantial factors.

### Recurrent thrombosis

**Figure 2.**
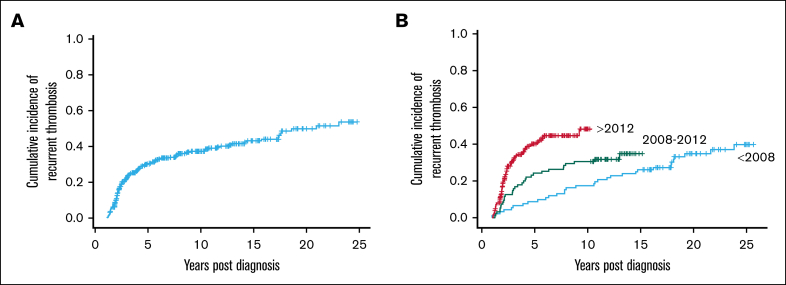
**Cumulative incidence of reccurent thrombosis.** Kaplan-Meier calculation of cumulative incidence of recurrent thrombosis from time of diagnosis in an overall cohort of patients with thrombotic APS (A), and calculation by era of diagnosis (B).

**Figure 3.**
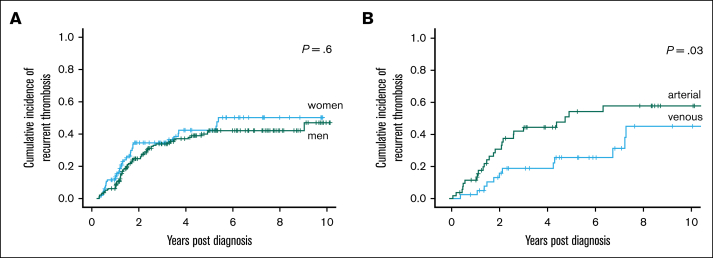
**Incidence of reccurent thrombosis based on sex and type of thrombosis.** Kaplan-Meier calculation of cumulative incidence of recurrent thrombosis from time of diagnosis, with comparison between men and women (A), and comparison between those with arterial thrombosis and VTE (B).

The Kaplan-Meier estimate of recurrence rate for all patients under follow-up was 55% at 25 years. However, this value encompasses diagnoses of thrombotic APS as far back as 25 years, and will be confounded by patients lost to follow-up over that time. To explore this, further analyses were performed to compare recurrence between various eras of diagnosis (Figure 2B). Complete follow-up was available for 313 of 500 patients who were diagnosed within the last decade of the study criteria (2012-2022). The median duration of follow-up for the 2012-2022 cohort was 1438 days. The Kaplan-Meier estimate of 10-year recurrence rate for this group was 46% (Figure 2B). Of note, recurrence largely occurred within the first 5 years of diagnoses, with very low recurrence rate after this point. There was overall no significance difference in recurrence between men and women (*P* = .6; Figure 3A). The 10-year probability of recurrence was higher in patients with arterial thrombosis compared with VTE (*P* = .03; Figure 3B).

The recurrence rates for the 95 of 500 patients diagnosed between 2008 and 2012, and 92 of 500 patients diagnosed before 2008 were lower overall than in the study period 2012-2022. This was largely expected due to death, loss of follow-up, and changes in diagnostic criteria and management of APS over time (Figure 2B).

### Probability of recurrent thrombosis according to aPL profile

**Figure 4.**
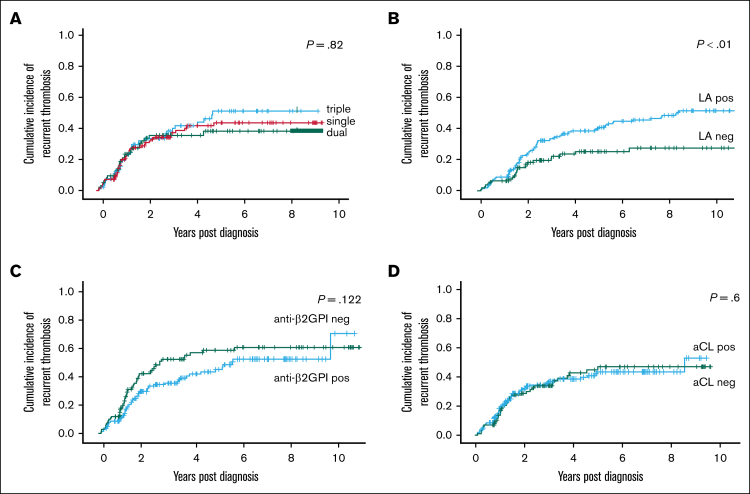
**Probability of recurrent thrombosis based on aPL profile.** Kaplan-Meier calculation of cumulative incidence of recurrent thrombosis from time of diagnosis based on aPL profile comparing single-, dual- and triple-positive groups (A), as well as each aPL (LA, aCL, anti-β2GPI) individually (B-D). neg, negative; pos, positive.

In the 2012-2022 cohort of patients, recurrent thrombosis occurred in 43 of 125 (34.4%) patients, 31 of 95 (32.6%) patients, and 37 of 93 (39.8%) patients with single-, dual- and triple-positive aPL, respectively. There was no difference in the probability of recurrent thrombosis based on the number of aPL (*P* = .82), especially in the first 3 years (Figure 4A). However, the probability of recurrent thrombosis was significantly higher in patients with LA (*P* < .01) compared with other antibodies (Figure 4B). No difference in the probability of recurrent thrombosis was seen with patients positive for anti-β2GPI antibodies (*P* = .122; Figure 4C) or aCL only, compared with other antibodies (*P* = .6; Figure 4D).

**Figure 5.**
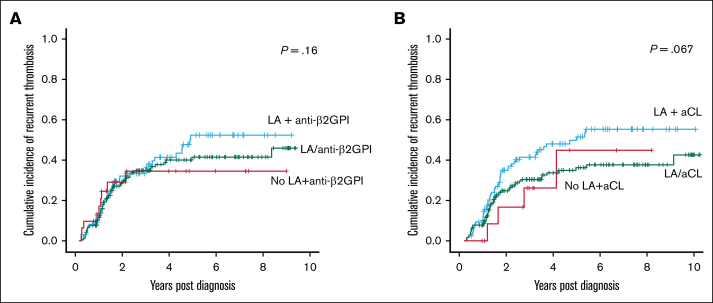
**Probability of recurrent thrombosis based on the presence of lupus anticoagulant.** Probability of recurrent thrombosis varies based on the combination of dual aPL. Kaplan-Meier calculation of cumulative incidence of recurrent thrombosis from time of diagnosis based on combinations of the 2 antibodies LA and anti-β2GPI (A), and LA and aCL (B).

Given the significant association of LA with recurrence, it was then assessed in combination with either aCL or anti-β2GPI. However, the recurrence rate was not altered by the combination of LA with anti-β2GPI or aCL (*P* = .16 combined with anti-β2GPI, and *P* = .06 combined with aCL), supporting the role of LA as the primary driver of thrombotic recurrence (Figure 5). Furthermore, rates of recurrence with isolated LA positivity and triple antibody are almost identical, again suggesting that it is LA that is the most potent predictor of thrombosis (compare Figure 4A, triple-positive curve, with Figure 4B, LA-positive curve).

### Management of recurrent thrombosis

Seventy four of 192 patients who developed recurrent thrombosis (38.5%) switched to different anticoagulation or increased the intensity of their existing anticoagulation. Of these 74 patients, 65 (87.9%) were on a DOAC, and all of these switched to a VKA. 9 out of 74 (12.1%) patients who had recurrent thrombosis were on VKA: 5 of 9 who developed recurrence while on VKA had a target international normalized ratio (INR) of 2.5 (2.0-3.0) and subsequently had their target INR increased to 3.5 (3.0-4.0). The remaining 4 patients (4 of 9) were switched to a DOAC. Of note, the 5 patients who had recurrence while on target INR of 2.5 were triple-positive, and the 4 patients who changed to a DOAC from VKA were non–triple-positive, and importantly these patients had time in the therapeutic range of <50% while on VKA. No second recurrences occurred following these described changes to anticoagulation within the study period.

### Use of hydroxychloroquine in thrombotic APS

Overall, 109 patients (21.8%) were treated with hydroxychloroquine (HCQ) and of these, 66 of 109 were given HCQ 200 mg once daily, whereas 43 of 109 (39.4%) were given 200 mg twice daily. Of the 109 patients on HCQ, in 14 (13%) patients the drug was started following the first thrombotic event, and in 40 (37%) patients following recurrent thrombosis. Of the remainder, HCQ was started with the concomitant diagnosis of other autoimmune disease (systemic lupus erythematosus and rheumatoid arthritis). It was found that patients with recurrent thrombosis were 13.8 times (95% CI, 2.39-26; *P* = .01) more likely to be started on HCQ compared with patients who had had a single thrombotic event, suggesting it is used as an adjunct to treatment in recurrent thrombosis. Of the 13 patients commenced on HCQ after the first thrombotic event, none developed recurrence within the study period.

### Bleeding events while on anticoagulation

The 10-year bleeding rate between 2012 and 2022 (major or clinically significant nonmajor bleeding) while on anticoagulation was 69 of 313 (22.0%) patients. Major bleeding occurred in 21 of 313 (6.7%) patients, and clinically significant nonmajor bleeding in 48 of 313 (15.3%) patients (as defined by the International Society for Haemostasis and Thrombosis Scientific Standardization committee).^12^^,^^13^ Of these, 57 of 69 (82.6%) occurred on VKA and 41 of 57 (71.9%) occurred on standard intensity VKA. Intracranial hemorrhage was the most common major bleeding event, comprising 10 of 21 (47.6%), followed by gastrointestinal bleeding comprising 6 of 21 (28.6%). Increasing age was the only factor significantly associated with major bleeding on MVA (OR, 1.05 [95% CI, 1.01-1.10]; *P* = .02).

## Discussion

We report the largest study to date assessing the thrombosis and bleeding events in a nationwide cohort of patients with thrombotic APS in the United Kingdom. This study has some important key findings; notably the high recurrent thrombotic rate of 46% within 10 years, principally in the first 5 years from diagnosis, the strong association between LA and recurrent thrombosis, and the relatively low bleeding rates in this cohort, 92% of whom were receiving antithrombotic therapy.

Our study provides valuable insight into the management of VTE and arterial thrombosis in APS nationally in the United Kingdom. Overall, we found that 95.1% of patients in the study who were triple-positive were placed on VKA and 4.9% on DOACS. This contrasts with 54.1% of patients on VKA and 24.8% on DOACs in non–triple-positive APS. Altogether this demonstrates that practice in the United Kingdom is largely in keeping with guidance for the highest risk APS cohort, but there remains a small group of patients who were triple-positive on DOACS, in some cases due to poor time in the therapeutic range INR.

Patients with arterial thrombosis and APS are an established high-risk group of patients with high rates of recurrence on standard anticoagulation or antiplatelet treatment, and no agreed consensus of management to date. In the Euro-Phospholipid Project Group, the most common arterial thrombotic manifestation was strokes, and in another Polish cohort study of 163 patients, arterial thrombosis manifested as stroke (65.3%), transient ischemic attacks (10.25%), and myocardial infarction (20.5%), which closely match the values in our own study (59.4%, 10.6%, and 30%, respectively).^14^ We did not find arterial thrombosis to occur more frequently in triple-positive disease, but it was associated with increasing age, presence of atherosclerotic risk factors, and dual aCL and anti-β2GPI positivity. Other studies have also implicated immunoglobulin G aCL antibodies in arterial thrombosis, and it is plausible that anti-β2GPI may further assist in identifying those at risk.^15^^,^^16^ When considering anticoagulation in this group, patients on DOACS consistently showed a significantly higher arterial recurrence.^10^ In this study, although not statistically significant, the use of a DOAC showed a trend toward an increased risk of recurrence (OR, 2.19 [95% CI, 0.52-11]; *P* = .3) compared with treatment with VKA (Table 2). We note that the UK practice has taken this concern into account, and 81.3% of patients with arterial thrombosis were on VKA and 6.7% on DOACs, with the remaining 11.9% of patients on alternative anticoagulants. There was also a greater preference for higher intensity INR with VKA in this group compared with VTE (27.7% vs 14.7%).

**Table 2.** **Factors included for the univariate analysis of probability of thrombosis recurrence in thrombotic APS**

Subgroup	OR for recurrence	95% CI	*P* value
**Age (y)**			
40			
40-60	0.58	0.28-1.22	.15
60+	1.23		.6
**Sex**			
Men			
Women	1.03	0.61-1.74	.9
**Anticoagulation at time of recurrence**			
VKA			
DOAC	2.19	0.02-0.37	.3
**Use of antiplatelet**			
No			
Yes	1.69	0.97-2.92	.062
**Ischemic heart disease**			
No			
Yes	2.36	0.97-2.92	.067
**Hypercholesterolemia**			
No			
Yes	1.95	0.90-4.22	.087
**Smoking**			
No			
Yes	1.48	0.73-2.93	.3
**High BMI**			
No			
Yes	1.94	0.88-4.29	.1
**Additional autoimmune disease**			
Hypothyroidism			
Sjogren syndrome	1.28	0.57-2.8	.5
SLE	5.75	1.28-40.1	**.035**
ITP	0.85		.6
**LA**			
No			
Yes	2.61	1.49-4.69	**<.01**
**Triple-positivity**			
No			
Yes	1.3	0.76-2.23	.3
**Double-positive: LA + aCL**			
No			
Yes	3.37	1.44-8.35	**.006**
**Double-positive: LA + anti-β2GPI**			
No			
Yes	1.21	0.67-7.4	.8
**Double-positive: aCL + anti-β2GPI**			
No			
Yes	1.35	0.16-1.68	**.004**

Bold values denote statistical significance (*P* < .05).

ITP, immune thrombocytopenia.

There was a significantly increased risk of recurrence in those with arterial thrombosis compared with VTE, confirming the need for more intensive antithrombotic regimens in this high-risk group (Figure 2B). Furthermore, there is little difference in the probability of recurrence between the patients who were non–triple- and triple-positive. This may be due to improved recognition of the highest risk patients who were triple-positive, resulting in more intensive anticoagulation and/or less frequent use of DOACs in patients who were triple-positive (10.6% vs 88.4%).

The role of combined antiplatelet and anticoagulant therapy for arterial thrombosis remains unclear. In our study, 68 (50.7%) patients with arterial thrombosis were on an antiplatelet treatment in addition to anticoagulation. A systematic review and meta-analysis of 13 studies including 719 patients showed that combined antiplatelet and warfarin is associated with a significant reduction in the risk of recurrent overall thrombosis (relative risk, 0.41 [95% CI, 0.20-0.85]) compared with single antiplatelet treatment.^17^ DOAC use was associated with a significant increase in the risk of recurrent arterial thrombosis, with a relative risk of 4.06 (95% CI, 1.33-12.40) when compared with single antiplatelet treatment. However, there was no significant difference in major bleeding among various antithrombotic strategies.^17^ As expected, atherosclerotic risk factors were more predominant in patients presenting with arterial thrombosis and associated with increased arterial thrombotic risk on MVA, highlighting the need for stringent cardiovascular risk factor management strategies, which were found to be suboptimal in the management of patients with APS.^18^

The most striking finding in this study was the confirmation of a high probability of recurrence of 46% at 10 years. In 2017, Pengo et al found the cumulative incidence of recurrent thromboembolic events to be 12.2% (95% CI, 9.6-14.8) after 1 year, 26.1% (95% CI, 22.3-29.9) after 5 years, and 44.2% (95% CI, 38.6-49.8) after 10 years.^2^ Similarly, in the 2015 Piedmont cohort study, the cumulative incidence of thrombotic recurrences was 12.5% at 1 year, 29% after 3 years, 38% after 5 years, and 49% after 10 years.^19^ An interesting finding in our study was low rates of thrombosis recurrence after 5 years from diagnosis. Randomized controlled trials have typically reviewed after shorter follow-up periods and may not have seen this phenomenon. Furthermore, we did not observe any second episodes of recurrence within the study period following the anticoagulation changes after initial recurrence. We speculate that could be due to improved standards of care, especially vascular risk reduction, effective modification of therapy, including return to VKA for high-risk (triple-positive) patients, continuation of anticoagulation and combination with HCQ in a significant number. Furthermore, it could also imply that there is a subgroup of patients with high thrombotic risk who might benefit from alternative or more intensive therapy from diagnosis.

Not all aPL profiles have the same contribution to risk of developing thrombosis, and LA positivity was defined as one of the high-risk aPL profiles according to the European Alliance of Associations for Rheumatology recommendations and Global Anti-Phospholipid Syndrome Score.^20^^,^^21^ In a meta-analysis of 16 441 patients from 30 studies that reported an OR of 6.14 (95% CI, 2.74-13.8; *P* < .001) for VTE associated with LA, compared with ORs of 1.46 and 1.61 for aCL and anti-β2GPI, respectively.^22^ For arterial thrombosis, the OR of LA was 3.58 (95% CI, 1.29-9.92; *P* = .01).^22^ Furthermore, LA has been identified as an independent risk factor for first thrombosis in aPL carriers. Our study is in agreement with this, and LA positivity had a significantly higher probability of recurrence compared with any other antibody in isolation (*P* < .01; Figure 4B). Furthermore, in our study, thrombosis free survival curves of isolated LA positivity and triple antibody are almost identical, suggesting that it is LA alone that is the most potent predictor of thrombosis (compare Figure 4A, triple-positive, with Figure 4B, LA-positive.) Whether this suggests that the presence of LA alone should warrant anticoagulation with VKA over DOAC remains to be determined, particularly in non–triple-positive APS; however, consideration must be given to the possible lack of standardization in testing of solid phase antibodies across various laboratories in the United Kingdom that may make aCL and anti-β2GPI less valuable in this context.

Interestingly, combined LA and aCL positivity (Figure 4B) had a trend toward an increased rate of recurrence (*P* = .067), which may delineate them as a higher risk group of patients with dual-positive thrombotic APS who may prove clinically valuable, though further studies may be required to confirm this.

Strategies for managing recurrence in APS are limited, but can include intensification of INR in the context of VKA, switch to low-molecular-weight heparin or addition of an antiplatelet treatment, or an immunomodulatory drug, in particular HCQ.^11^^,^^23^ Although not established practice throughout the United Kingdom, HCQ has demonstrated antithrombotic effects in in vivo and in vitro studies.^24^ Due to the small number of patients started on HCQ after a thrombotic event at APS diagnosis in this study (n = 13), we are unable to accurately assess its impact on recurrence; however, none of these 13 patients went on to develop recurrence during follow-up. We did however find that patients with recurrent thrombosis were 13.8 times more likely to receive HCQ compared with patients without recurrent thrombosis.^24^^,^^25^ HCQ may be particularly of use in those with coexistent systemic lupus erythematosus, a group that this study identified as having a significantly increased probability of recurrence.^26^

Clinically significant bleeding was experienced by 22.0%, with 6.7% of patients having major bleeding. These figures are lower than the rate in large meta-analyses of anticoagulation for VTE that found the 5-year cumulative incidence of major bleeding with VKAs in the general population to be 6.3% (95% CI, 3.6%-10.0%). This may reflect underreporting leading to bias or be due to the underlying prothrombotic phenotype of this cohort.^27^ Recent work by a single-center group documented a major bleeding rate of 22.8%; however, this study included 24.9% of patients on combined antithrombotic therapy as well as 41.9% on high-intensity anticoagulation with target INR >3.^28^

The strengths of the present study are that it is the largest study of APS in the United Kingdom documenting real-world data over a 10-year period representative of practice in the United Kingdom in both tertiary and nontertiary centers. It had similar overall response rates to previously published data, but provides more long-term follow-up and detailed assessment of recurrence. This study has several limitations. Its retrospective, observational design limits causal inference and introduces potential confounding by indication, as treatment choices were influenced by clinician judgment and evolving guidelines. Although data were collected using a predesigned electronic case report form to maintain uniformity, data were obtained from the routine clinical records across multiple centers, resulting in heterogeneity in follow-up, imaging practices, and laboratory assays, particularly for aPL testing, which may have affected antibody classification. LA testing may have been influenced by concurrent anticoagulation, leading to possible misclassification. Recurrent thrombotic and bleeding events may have been under-ascertained if patients presented to other institutions and not reported to the managing center. Follow-up duration varied and, as noted, longer-term recurrence estimates may be affected by loss to follow-up. Finally, subgroup analyses, particularly adjunctive therapies such as HCQ, were limited by small numbers and should be considered exploratory.

In conclusion, in the United Kingdom, most patients with APS with high-risk VTE and arterial thrombosis were treated with VKA. However, the rate of recurrent thrombosis remained high. Although the rate of major bleeding is low, increasing age was significantly associated with major bleeding. Taken together, there is an outstandingly high thrombotic risk in APS, and identifying patients with APS at higher risk of recurrent thrombosis is still an unsolved issue. This unacceptably high rate of recurrent thrombosis may further support the need to unravel and target alternative pathogenic mechanisms of thrombosis in APS in addition to anticoagulation to prevent recurrent thrombosis. Finally, LA has shown a significant association with recurrent thrombosis, with both aCL and anti-β2GPI having minimal impact. This highlights the importance of appropriate testing of LA in patients with suspected APS that is frequently affected by anticoagulation.

Conflict-of-interest disclosure: C.C.-T. reports speaker fees and honoraria from Roche, Chugai, Sobi, and Novo Nordisk; and research funding from AstraZeneca. A.J.D. reports speaker fees from Viatris. M.L. reports grant/research support from Bayer and Commonwealth Serum Laboratories Behring; consultant fees from Shire, LFB, Chugai, Roche, Sobi, Octapharma, Baxter, Bayer, Pfizer, and Commonwealth Serum Laboratories; and was part of speaker bureau for Pfizer, Bayer, and Octapharma. D.J.A. reports speaker fees from Werfen; and travel grants from Stago and Sysmex. The remaining authors declare no competing financial interests.

A complete list of the HaemSTAR Study Collaborators appears in “Appendix.”

## Appendix

The HaemSTAR Study Collaborators are: Giulia Simini, Imperial College Healthcare NHS Trust; Lara Roberts and Amna Gameil, Kings College London; Gillian Lamb, Kay Simpson, and Nini Aung, North Tees and Hartlepool NHS Foundation Trust; Li Yuan Chan, Royal Surrey County Hospital NHS Foundation Trust; Emily Booth, Ann Benton, James Duffell, and Kieron Power, Wales/Cardiff/Swansea Bay UHB; Saniya Dhawan and Izabela James, University Hospital Southampton; Sarah Lewis, Jessica Anderson, and Sahil Bhagat, Cardiff and Vale University Health Board; Mohammed Altohami, Amjad Hmaid, Dina Abuqamar, Pedro Goncalves, Zunaid Chunara, Styliani Salta, and Amy Webster, University Hospital of Leicester NHS Trust; Sarah Challenor, University Hospital Plymouth NHS Trust; Pip Nicholson, David Sutton, and Richard Buka, University Hospital North Midlands NHS Trust; Angel Joseph and Victor Ling, Wigan and Leigh NHS Foundation Trust; Jacob Nkem, NHS Greater Glasgow; Kat Moss and Andrew Ross, St Helens & Knowsley Teaching Hospitals NHS Trust; Emily Millen and Alexander Bashford, University Hospitals Nottingham; Prajakta Pardeshi and Philip Mounter, County Durham and Darlington Trust; Juan Tan and Cecilia Gyansah, Norfolk and Norwich University NHS Trust; Tina Dutt, Samuel Badu, and Francesca Murphy, University Hospitals of Liverpool; Alexander Langridge, Mohammad Osama, Paula Glancy, and Shikha Chattree, SRH and South Tyneside Hospitals; Alexander Langridge, Kathryn Musgrave, and Beatrice Likupe, Newcastle Upon Hospitals NHS Trust; and Alison Delaney, Sheffield University Hospitals NHS Trust.
